# NT-3 Combined with TGF-β Signaling Pathway Enhance the Repair of Spinal Cord Injury by Inhibiting Glial Scar Formation and Promoting Axonal Regeneration

**DOI:** 10.1007/s12033-023-00781-4

**Published:** 2023-06-15

**Authors:** Taibang Chen, Xiaoqing He, Jing Wang, Di Du, Yongqing Xu

**Affiliations:** Department of Orthopedics, No. 920 Hospital of PLA Joint Logistics Support Force, No. 212 Daguanlu, Kunming, Yunnan 650000 China

**Keywords:** NT-3, Spinal cord injury, TGF-β, Glial scar

## Abstract

This study investigated the mechanism of neurotrophin-3 (NT-3) in promoting spinal cord injury repair through the transforming growth factor-beta (TGF-β) signaling pathway. A mouse model of spinal cord injury was established. Forty C57BL/6J mice were randomized into model, NT-3, NT-3 + TGF-β1 and NT-3 + LY364947 groups. The Basso–Beattie–Bresnahan (BBB) scores of the NT-3 and NT-3 + LY364947 groups were significantly higher than the model group. The BBB score of the NT-3 + TGF-β1 group was significantly lower than NT-3 group. Hematoxylin-eosin staining and transmission electron microscopy showed reduction in myelin sheath injury, more myelinated nerve fibers in the middle section of the catheter, and relatively higher density and more neatly arranged regenerated axons in the NT-3 and NT-3 + LY364947 groups compared with the model and NT-3 + TGF-β1 groups. Immunofluorescence, TUNEL and Western blot analysis showed that compared with model group, the NEUN expression increased, and the apoptosis and Col IV, LN, CSPG, tenascin-C, Sema 3 A, EphB2 and Smad2/3 protein expression decreased significantly in the NT-3 and NT-3 + LY364947 groups; the condition was reversed in the NT-3 + TGF-β1 group compared with the NT-3 group. NT-3 combined with TGF-β signaling pathway promotes astrocyte differentiation, reduces axon regeneration inhibitory molecules, apoptosis and glial scar formation, promotes axon regeneration, and improves spinal cord injury.

## Introduction

Stem cell transplantation is considered to be one of the most promising methods for the treatment of spinal cord injury [1–5]. However, the repair effect of simple neural stem/progenitor cells (NSPCs) transplantation is far from expected, and there are many influencing factors [6]. The transplantation of NSPCs is also limited by the source of stem cells and medical ethics. Therefore, activating endogenous neural stem cells to proliferate and differentiate has gradually attracted people’s attention [7, 8].

There is an internal environment in the spinal cord that strongly inhibits the differentiation and survival of endogenous neural stem cells and remyelination of neuronal axons after spinal cord injury [9, 10]. Neurotrophin-3 (NT-3), a member of the nerve growth factor family of neurotrophins, can promote the survival and differentiation of a variety of central and peripheral neurons, regulate neuronal synaptic activity, and play an important role in the development and maturation of the nervous system. It can promote the repair of nerve injury by protecting neurons and promoting axon regeneration [11]. After spinal cord injury, fibroblasts migrate to the injury center to form fibroblast clusters, which are wrapped by reactive astrocytes to form glial scars to prevent secondary spinal cord injury. Glial scar is a physical barrier to re-establish nerve conduction pathway and form functional synapse. Fibroblast clusters express a variety of axon growth inhibitory molecules, which is the main obstacle to axon regeneration.

Transforming growth factor-beta 1 (TGF-β1) is an effective fibrotic factor, which participates in the proliferation of fibroblasts and the formation of cell clusters. Blocking this factor can inhibit the proliferation of fibroblasts and promote the regeneration of axons of fibrous scar neurons [12, 13]. This study explored the molecular mechanism of NT-3 combined with TGF-β1 signaling pathway in the repair of spinal cord injury in vitro and in vivo.

## Materials and Methods

### Ethical statement

This study was approved by the Institutional of Animal Care and Use Committee of the authors’ institution, and conducted in compliance with the guidelines.

### Mouse Model of Spinal Cord Injury

C57BL/6J specific pathogen-free (SPF) female mice (18–22 g) were purchased from SPF (Beijing) Biotechnology Co., Ltd., China. After anesthesia, the mice were fixed, and the back was shaved and disinfected. The position of the T10-12 thoracic vertebrae was determined. The skin was cut carefully and the muscles on both sides of the thoracic vertebrae were separated. The position of T10-12 thoracic vertebrae was determined again, and the muscles near the lamina was carefully cut to expose the T10-12 thoracic vertebrae. The lamina was peeled off carefully with micro hemostatic forceps to avoid accidental injury to the spinal cord. A Kirschner wire with a diameter of 2 mm and a weight of 5 g was used. It was placed in the middle of the spinal cord with a 4 mm hollow tube, and smashed at a fixed point of 4.5 cm. The reaction of the mice was observed and recorded. Twitch and spasm of the hindlimbs and tail flick indicated that the model was successfully established. The muscles on the both sides of the exposed spinal cord were sutured with a 3 − 0 suture. 3% hydrogen peroxide disinfectant was dripped to prevent wound infection. The excess fluid was wiped, and the outer skin was sutured. After suture, the outer skin was disinfected, and the mice were placed on a thermal insulation pad to wait for awakening.

### Experimental Grouping and Drug Administration

The mice were randomly allocated into model, model + NT-3 (NT-3), model + NT-3 combined with TGF-β1 (NT-3 + TGF-β1) and model + NT-3 + LY364947 (NT-3 + LY364947) groups. There were 10 mice in each group. The NT-3 group was administered with NT-3, the NT-3 + TGF-β1 group was administerd with NT-3 + TGF-β1, and the NT-3 + LY364947 group was administered with NT-3 + LY364947. NT-3 was purchased from Cloud-Clone Corp., Texas, USA. TGF-β1 was from Solarbio, Beijing, China. LY364947 (CAS: 396129-53-6) was from GlpBio, CA, USA. The concentration of each drug was 20 ng/ml, and the mice in each group were administered at the injured site half an hour after the modeling. As the administration time was short, and the administration site was special, so it was not sutured first. The drug was dripped at a dosage of 10 ml/kg on the injured site, let it fully absorbed, and then sutured.

The reason for using female mice was that after the spinal cord injury operation, the hindlimbs of the mice became flaccid and paralyzed, there was an obstacle in urination, and they required artificial assistance in urination. The urethra of female mice is short and the mice are easy to urinate, while the urethra of male mice is narrow and long, causing urination difficult and leading to a higher mortality rate.

Postoperative care: Antibiotics were injected daily after the operation, and urination were performed twice a day. After modeling, the mice were raised individually in a single cage, and the padding was changed every 2 days to ensure the cage environment was appropriate.

### Functional Evaluation by Basso–Beattie–Bresnahan (BBB) Score

On day 3 and 7 after operation, functional evaluation of all animals were performed using the modified standard BBB score (Table 1). On the 14th day after scoring, the mice in each group were euthanized by excessive anesthesia, and the spinal cord tissue was collected.

**Table 1:** Functional evaluation by Basso–Beattie–Bresnahan (BBB) score

Item	Score
No hindlimb movement	0
Spastic, uncontrolled unilateral hindlimb movements	1–2
Spastic, uncontrolled bilateral hindlimb movements	3–5
Controlled unilateral hindlimb movements	6–7
Controlled bilateral hindlimb movements	8–9
Assisted walking with unilateral hindlimb	10–12
Assisted walking with bilateral hindlimbs	13–21

.

### Hematoxylin-Eosin (HE) Staining

The spinal cord tissues were washed with running water. They were dehydrated with 70%, 80% and 90% ethanol solutions and the mixture of pure alcohol and xylene for 15 min. Then, they were treated with xylene I for 15 min, and xylene II for 15 min (until clear), embedded with paraffin and cut into slices. The paraffin slices were baked, dewaxed and hydrated. They were stained with hematoxylin for 3 min, differentiated with hydrochloric acid ethanol for 15 s, and treated with bluing solution for 15 s. Then, they were washed, stained with eosin for 3 min, washed, dehydrated, cleared, mounted, and examined under the microscope (CX41, Olympus Corp., Shinjuku, Tokyo, Japan).

### Immunofluorescence Detection of the Neuronal Nuclei (NeuN)/Glial Fibrillary Acidic Protein (GFAP) Expression in Spinal Cord Injury

The paraffin-embedded tissue sections were dewaxed, incubated in the citrate buffer for antigen retrieval, heated for 2 min in a pressure cooker and cooled down naturally. After washing with phosphate-buffered saline (PBS), the sections were treated with 0.5% Triton X-100 at room temperature. The sections were washed in PBS (5 min × 3). After blocking in 5% bovine serum albumin (BSA) at 37 °C for 30 min, the sections were incubated with the primary antibodies [anti-NeuN (1:200) (Cell Signaling Technology, Danvers, MA, USA), anti-GFAP (1:300) (Abcam PLC, Cambridge, UK)] at 4 °C overnight. They were washed, added with fluorescent secondary antibody Cy3 (1:200) (Beyotime Biotechnology, Shanghai, China), and incubated at 37 °C for 30 min. Then, the sections were stained with 4’,6-diamidino-2-phenylindole (DAPI) solution in dark for 5 min. The excess DAPI was washed with PBS, and with tap water for 1 min. The sections were mounted and observed under the microscope (CKX53, Olympus Corp.).

### Terminal Deoxynucleotidyl Transferase dUTP Nick-End Labeling (TUNEL) Detection of Apoptosis in Spinal Cord Injury Area

The slices were routinely dewaxed, hydrated, and transferred into a wet box. Proteinase K (50 µg/ml) solution was added and incubated at 37 °C for 30 min. Then, they were washed with PBS for three times (5 min each time). TUNEL test solution (Beyotime Biotechnology) was added and incubated at 37 °C in dark for 1 h. The slices were mounted and observed under the fluorescence microscope (CKX53, Olympus Corp.).

### Transmission Electron Microscope (TEM) Experiment

The samples were fixed in 2.5% glutaraldehyde for transmission electron microscopy. They were embedded with acetone embedding solution and solidified in an oven. They were cut into 70 nm slices, subjected to 3% uranium acetate-lead citrate double staining, and examined under the JEOL JEM-1230 (80 kV) TEM (JEOL Ltd., Akishima, Tokyo, Japan).

### Western Blot

The tissues were lysed on ice for 30 min. They were centrifuged at 4 °C (12000 rpm) for 15 min, the supernatant was collected, and the proteins were extracted. The proteins (50 µg/lane) were separated using sodium dodecyl sulfate-polyacrylamide gel electrophoresis (SDS-PAGE) (DYY-6C, Beijing Liuyi Biotechnology Co., Ltd., China) and transferred to the polyvinylidene fluoride (PVDF) membrane (MilliporeSigma, Burlington, MA, USA). The membrane was washed and treated with blocking buffer. The primary antibodies against collagen (Col) (IV) (1:1000), semaphorin (Sema) 3A (1:1000), ephrin type-B receptor 2 (EphB2) (1:1000), mothers against decapentaplegic homolog 2/3 (Smad2/3) (1:1000) (Affinity Biosciences, Cincinnati, OH, USA), laminin (LN) (1:1000), chondroitin sulfate proteoglycan (CSPG) (1:1000) (Abcam PLC), and tenascin-C (1:1000) (Proteintech Group, Rosemont, IL, USA) were added and incubated at 4 °C overnight. The membrane was rinsed, the secondary antibody (1:2000) (ZSBG-Bio, Beijing, China) was added, and they were incubated at room temperature for 1 h. β-actin (1:2000) (ZSGB-Bio) was used as internal control. An enhanced chemiluminescence (ECL) kit (Thermo Fisher Scientific, Waltham, MA, USA) was used for immunoblotting, and “Quantity one” software (Bio-Rad Laboratories, Hercules, CA, USA) was used for quantification.

### Statistical Analyses

The statistical tests were conducted using SPSS 20.0 software (IBM Corp., Armonk, NY, USA). All experiments were repeated three times. The quantitative values between two groups were compared with independent sample t-test, and the differences among groups were analyzed using one-way analysis of variance (ANOVA). Student–Newman–Keuls (S-N-K) method was used for pairwise comparison. *P* < 0.05 was considered statistically significant.

## Results

### BBB Score

**Fig. 1:**
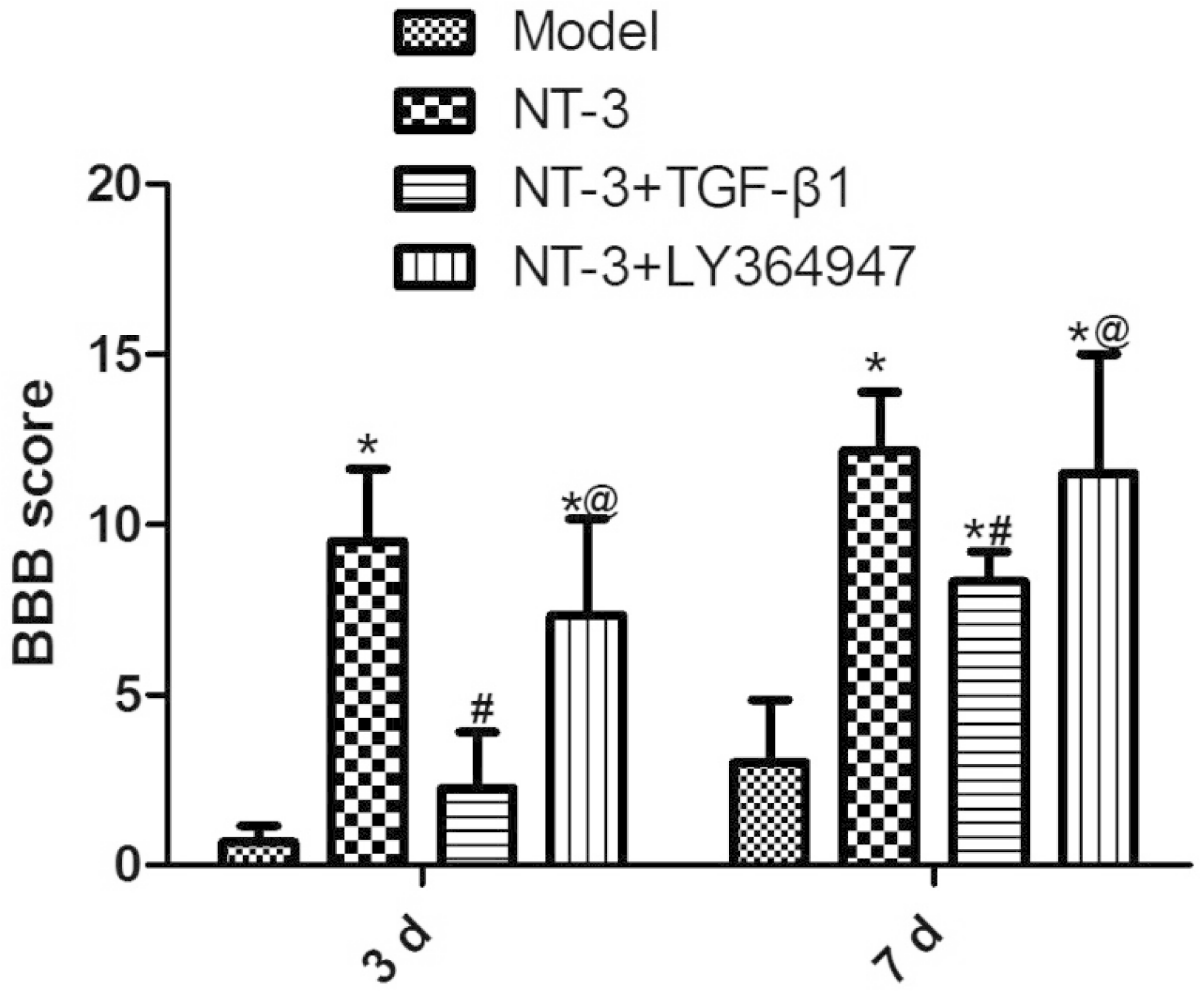
BBB scores in each group of mice with spinal cord injury at day 3 and day 7 after operation. *P < 0.05 vs. model group; ^#^P < 0.05 vs. NT-3 group; ^@^P < 0.05 vs. NT-3 + TGF-β1 group. Abbreviations: BBB: Basso–Beattie–Bresnahan, NT-3: neurotrophin-3, TGF-β1: transforming growth factor-beta 1.

The BBB score of each group increased to varying degrees from 3–7 days after operation. At the two time points, the BBB scores of the NT-3 and NT-3 + LY364947 groups were significantly higher than that of the model group (P < 0.05), while the BBB score of the NT-3 + TGF-β1 group was significantly lower than that of the NT-3 group (P < 0.05) (Fig. 1).

### Pathomorphological Observation of the Spinal Cord Injury Area

**Fig. 2:**
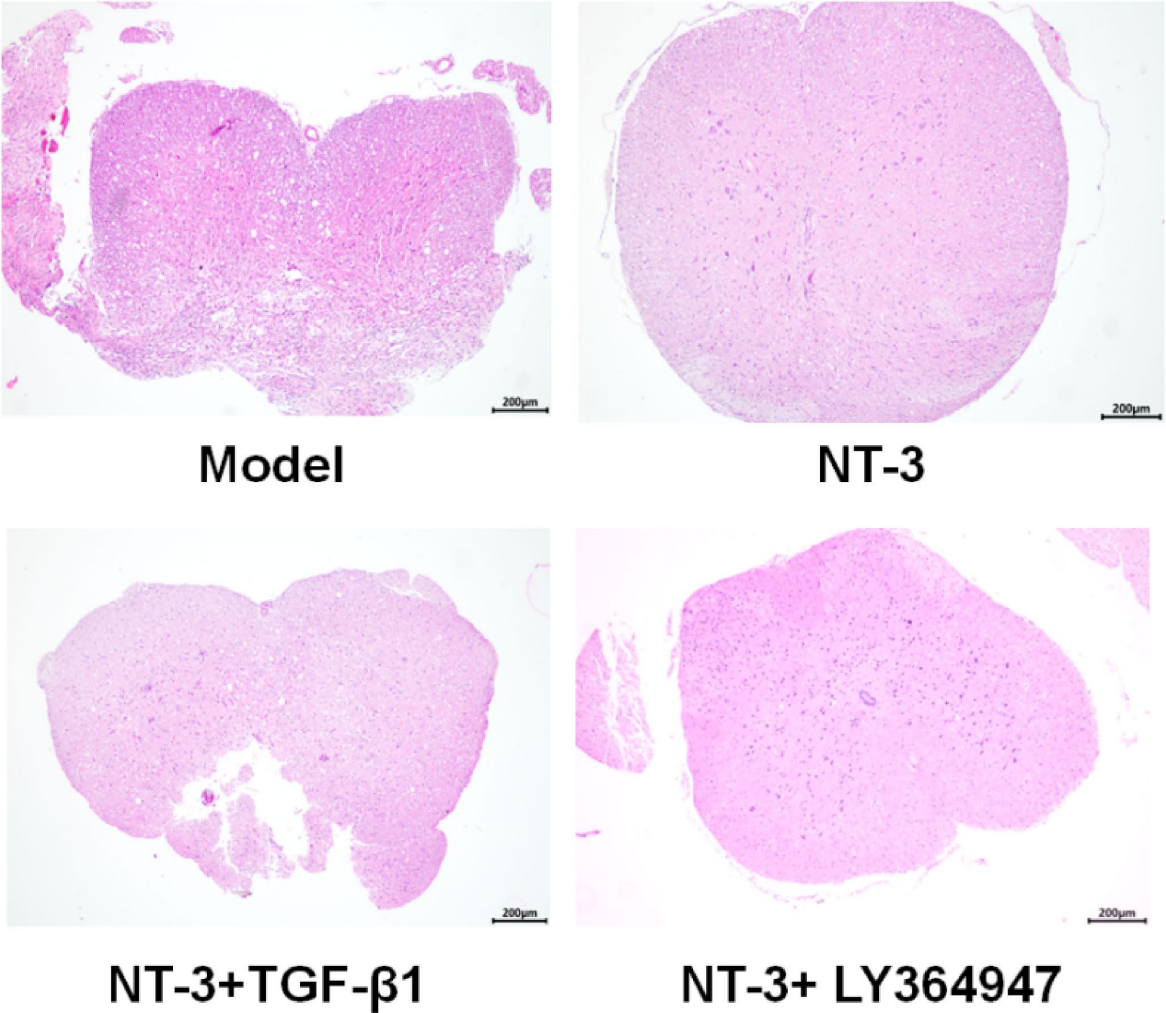
Histopathological morphology of spinal cord tissue in each group of mice (HE staining) (40x). In model and NT-3 + TGF-β1 groups, the nucleus of spinal cord nerve was fragmented, the tissue structure was loose with a large number of vacuolar degeneration after spinal cord injury. In NT-3 and NT-3 + LY364947 groups, the above pathology was improved, the structure was dense and the number of cells increased. Abbreviations: HE: hematoxylin-eosin; NT-3: neurotrophin-3, TGF-β1: transforming growth factor-beta 1.

As shown in Fig. 2, in the model and NT-3 + TGF-β1 groups, the nucleus of the spinal cord nerve was fragmented, the tissue structure was loose, edematous, the boundary between the gray matter and the white matter was blurred, and a large number of vacuolar degeneration were seen after spinal cord injury. Compared with the model group, the structure was dense, the number of cells was increased, small and poorly differentiated nucleated cells appeared, and the pathological injury of the spinal cord was significantly improved in the NT-3 and NT-3 + LY364947 groups.

### Apoptosis in the Spinal Cord Injury Area

**Fig. 3:**
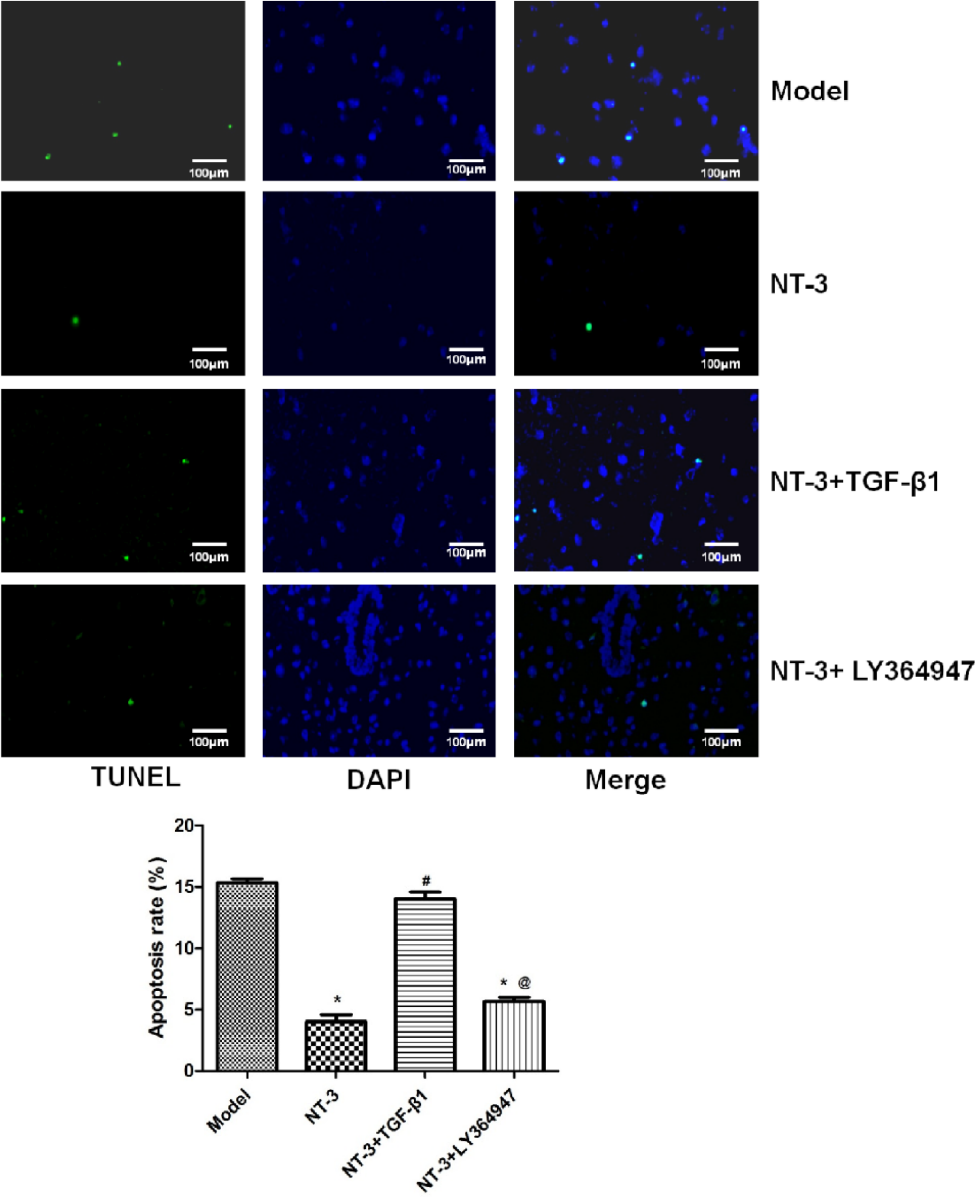
Apoptosis in spinal cord tissue in each group of mice detected by TUNEL. *P < 0.05 vs. model group; ^#^P < 0.05 vs. NT-3 group; ^@^P < 0.05 vs. NT-3 + TGF-β1 group. Abbreviations: TUNEL: terminal deoxynucleotidyl transferase dUTP nick-end labeling, NT-3: neurotrophin-3, TGF-β1: transforming growth factor-beta 1.

Compared with the model group, the apoptosis in the NT-3 and NT-3 + LY364947 groups decreased significantly (P < 0.05). Compared with the NT-3 group, the apoptosis in the NT-3 + TGF-β1 group increased significantly (P < 0.05) (Fig. 3).

### Ultrastructure Observed by Transmission Electron Microscope

**Fig. 4:**
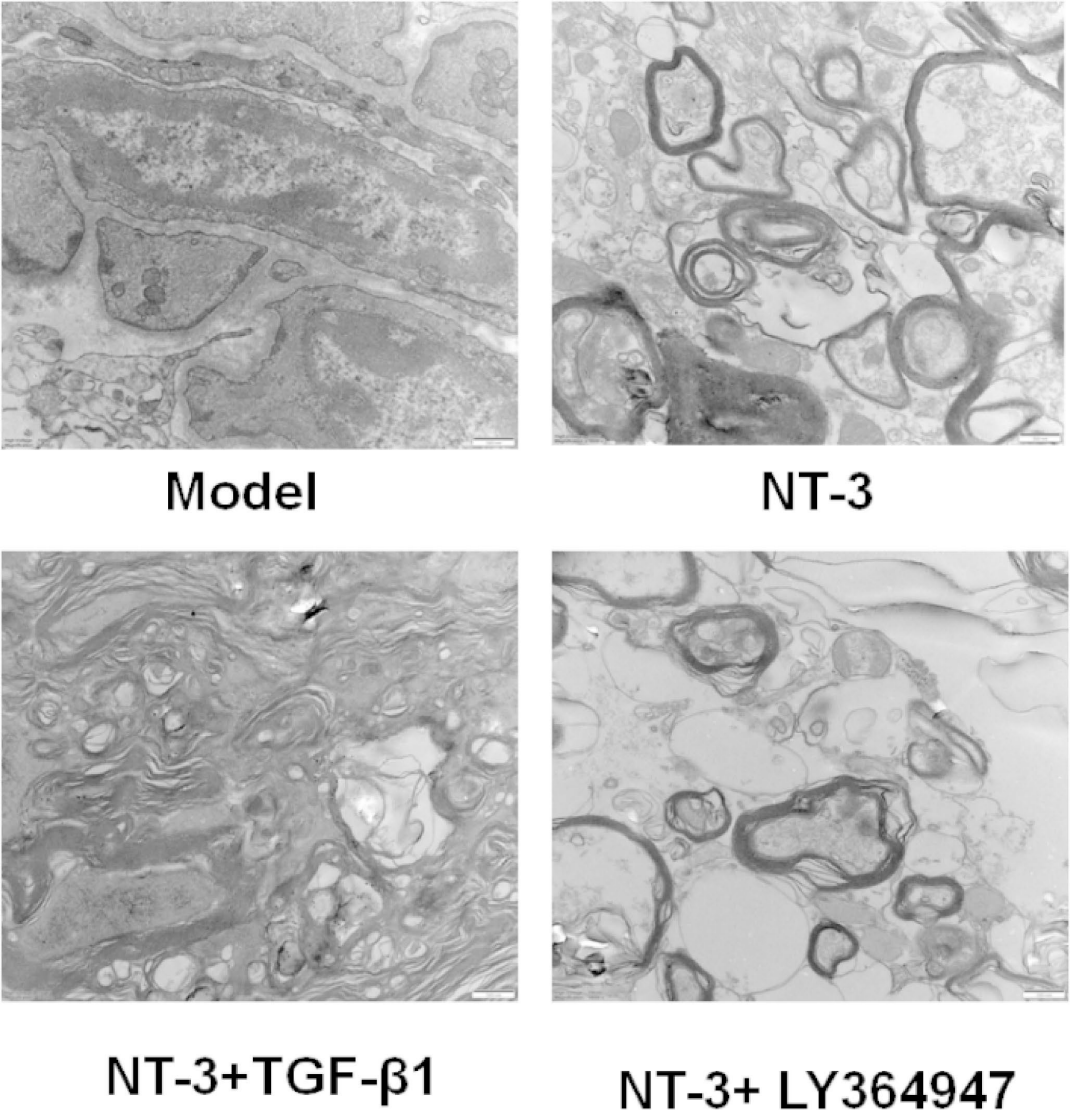
Myelin sheath in spinal cord tissue in each group of mice detected by TEM. Abbreviations: TEM: transmission electron microscope, NT-3: neurotrophin-3, TGF-β1: transforming growth factor-beta 1.

In the model and NT-3 + TGF-β1 groups, the myelin sheath was loose and broken seriously, a large number of fibroblasts, astrocytes and collagen fibers were densely arranged in the injured area, forming glial scar tissue, and no new axons were found. In the NT-3 and NT-3 + LY364947 groups, the myelin sheath injury was reduced, and more myelinated nerve fibers could be seen in the middle section of the catheter. The density of the regenerated axons in the latter was relatively higher, the arrangement was more neatly, the thickness of the myelin sheath varied, and they were in continuous formation and thickening (Fig. 4).

### NeuN/GFAP Expression

**Fig. 5:**
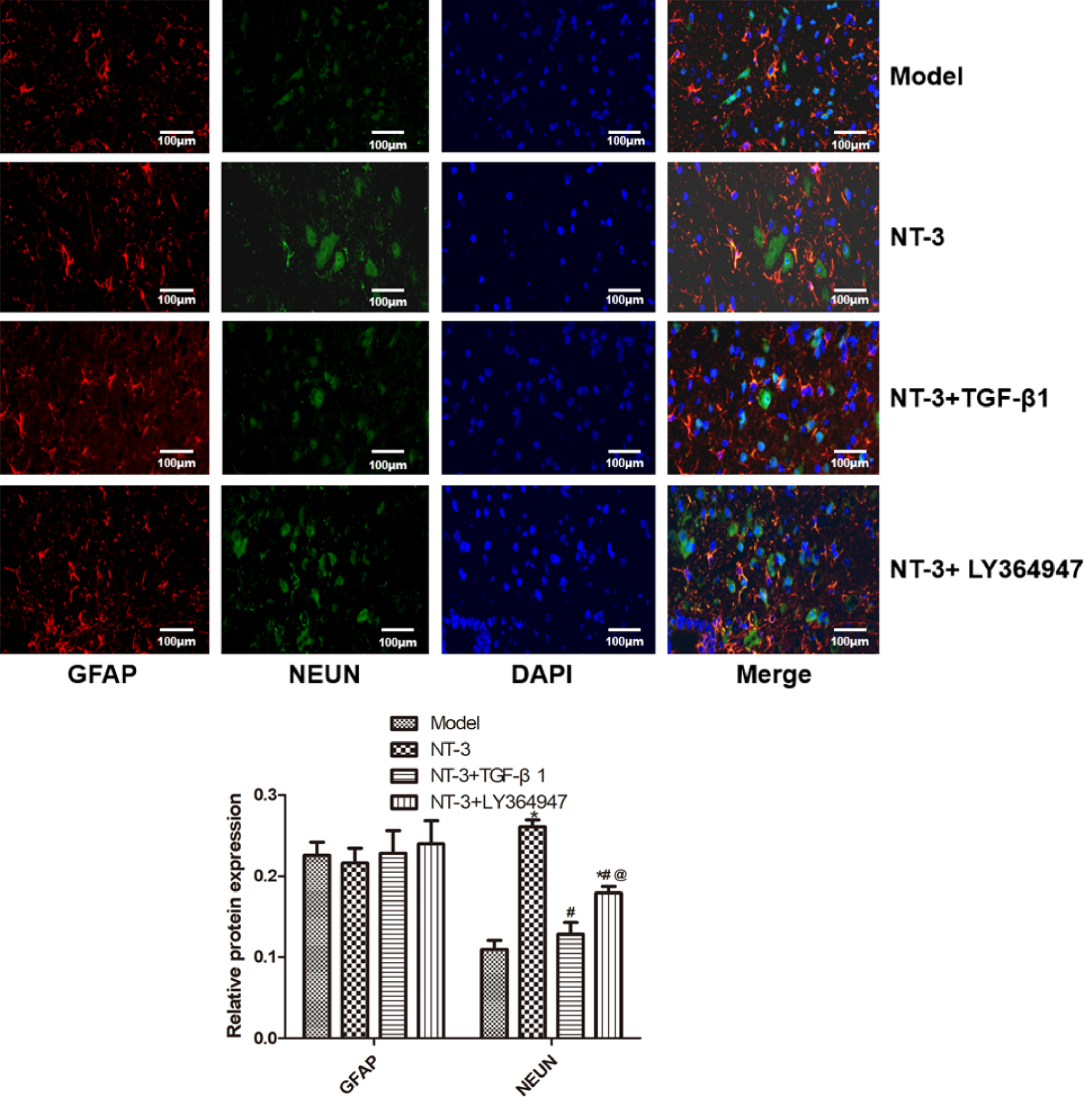
NeuN/GFAP expression in spinal cord tissue of mice in each group detected by double immunofluorescence staining. *P < 0.05 vs. model group; ^#^P < 0.05 vs. NT-3 group; ^@^P < 0.05 vs. NT-3 + TGF-β1 group. Abbreviations: NeuN: neuronal nuclei, GFAP: glial fibrillary acidic protein; NT-3:  neurotrophin-3, TGF-β1: transforming growth factor-beta 1.

Compared with the model group, the NeuN expression in the NT-3 and NT-3 + LY364947 groups increased significantly (P < 0.05). Compared with the NT-3 group, the NeuN expression in the NT-3 + TGF-β1 group decreased significantly (P < 0.05). There was no significant difference in GFAP expression among groups (Fig. 5).

### Western Blot

**Fig. 6:**
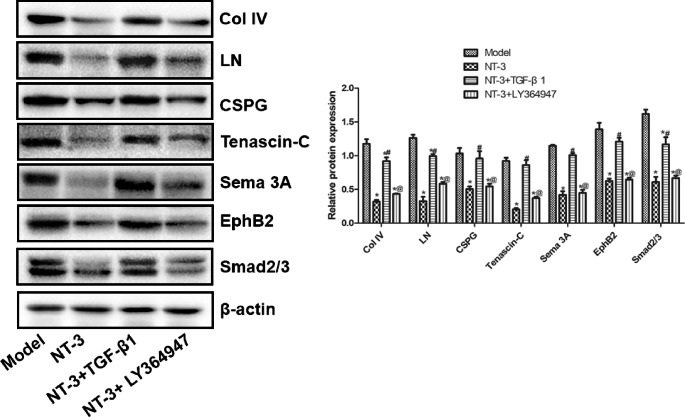
Col IV, LN, CSPG, tenascin-C, Sema 3A, EphB2, smad2/3 protein expression in spinal cord tissue of mice in each group detected by Western blot. *P < 0.05 vs. model group; ^#^P < 0.05 vs. NT-3 group; ^@^P < 0.05 vs. NT-3 + TGF-β1 group. Abbreviations: Col IV: collagen IV, LN: laminin, CSPG: chondroitin sulfate proteoglycan, Sema 3A: semaphorin 3A, EphB2: ephrin type-B receptor 2, smad2/3: mothers against decapentaplegic homolog 2/3, NT-3: neurotrophin-3, TGF-β1: transforming growth factor-beta 1.

The expression levels of Col IV, LN, CSPG, tenascin-C, Sema 3A, EphB2 and Smad2/3 proteins in the NT-3 and NT-3 + LY364947 groups were significantly lower than that of the model group (P < 0.05), while these proteins in the NT-3 + TGF-β1 group increased significantly compared with that of the NT-3 group (P < 0.05) (Fig. 6).

## Discussion

Spinal cord injury is a common traumatic neurological condition, which is difficult to reverse. The treatment time and severity of spinal cord injury determine the prognosis of patients. At present, neural stem cell transplantation is favored by the majority of scientific researchers among many treatment schemes for spinal cord injury. Neural stem cells have the potential of self-renewal, proliferation, migration and multidirectional differentiation. The differentiated neurons can replace the dead and lost neurons, form new synaptic connections with the surrounding normal neurons and reconstruct conduction pathways [14, 15]. Due to the limited availability of exogenous neural stem cells, and that they are prone to immune rejection and ethical problems, which seriously restrict their clinical transformation, how to activate endogenous neural stem cells and promote their growth, proliferation and differentiation has become a hot topic for scientific researchers recently [16, 17]. The proliferation of endogenous neural stem cells is very limited under normal conditions. Only when the spinal cord is injured, it will be activated and proliferate in large numbers, and differentiate in different directions [18, 19]. Endogenous neural stem cells can proliferate and differentiate into different types of mature tissue cells in different environments [20, 21]. Therefore, when the internal environment changes after spinal cord injury, the activation of microglia, the scar formed by astrocyte proliferation and the production of related axon growth inhibitory factors lead to a small number of proliferative endogenous neural stem cells [22, 23]. This is the most prominent problem in the treatment of spinal cord injury by endogenous neural stem cells.

This study explored the effect of NT-3 combined with TGF-β signaling pathway on the repair of spinal cord injury. Glial scar formed by reactive proliferation of astrocytes is a key factor hindering axon regeneration [24]. TGF-β is closely related to scar formation. TGF-β signaling pathway is activated after spinal cord injury, TGF-β level in glial cells, neurons and other cells in spinal cord tissue increases and it can promote glial scar hyperplasia by regulating the transcription of target genes. In addition to forming a mechanical barrier, glial scar can also produce inhibitory molecules to inhibit the growth of axons, which will eventually lead to serious consequences of permanent loss of nerve function. Inhibiting TGF-β signaling pathway to reduce the production of glial scar is of great significance for the repair of spinal cord injury [25].

NT-3 is a small molecular weight basic protein, which belongs to the nerve growth factor gene family. It can regulate the development, growth, differentiation and regeneration of neurons. Studies found that NT-3 can promote the differentiation of neural stem cells into neurons and oligodendrocytes, while there is relatively little differentiation into astrocytes [26–28]. Ramu et al. found that after 4 weeks of NT-3 treatment, the injured contralateral cerebral cortex, thalamus, caudate nucleus, hippocampus and other peripheral gray matter of rats showed strong signals [29], Hanna-Mitchell et al. found that NT-3 reduced neuronal apoptosis and promoted axon growth in the central nervous system [30].

BBB score can well evaluate the motor function of the mice hindlimb by analyzing the range of motion, coordination and gait stability of the hindlimb joints. It is the most widely used evaluation method for the recovery of spinal cord injury at present [31]. LY364947 (HTS 466284) is a potent adenosine triphosphate (ATP)-competitive inhibitor of transforming growth factor beta receptor I (TGFβR-I). This study showed that compared with the model group, the hindlimb function of the mice in the NT-3 and NT-3 + Ly364947 groups began to recover gradually, and the BBB score increased significantly, while the BBB score of the NT-3 + TGF-β1 group decreased significantly compared with that of the NT-3 group.

In the model and NT-3 + TGF-β1 groups, the nucleus of the spinal cord nerve was fragmented, the tissue structure was loose with a large number of vacuolar degeneration after spinal cord injury. Compared with the model group, the structure was dense, the number of cells was increased, small and poorly differentiated nucleated cells appeared, and the pathological injury of the spinal cord of mice was significantly reduced in the NT-3 and NT-3 + Ly364947 groups. The TEM results showed that in the model and NT-3 + TGF-β1 groups, the myelin sheath was loose and broken seriously, a large number of fibroblasts, astrocytes and collagen fibers were densely arranged in the injured area, forming glial scar tissue, and no new axons were found. In the NT-3 and NT-3 + Ly364947 groups, the myelin sheath injury was reduced and more myelinated nerve fibers were seen in the middle section of the catheter. The density of the regenerated axons in the latter was relatively higher and the arrangement was more neatly. Apoptosis plays a very important role in secondary spinal cord injury [32]. The TUNEL results showed that compared with the model group, the apoptosis in the NT-3 and NT-3 + Ly364947 groups decreased significantly, while the apoptosis in the NT-3 + TGF-β1 group increased significantly compared with that of the NT-3 group. These results suggested that NT-3 combined with the TGF-β signaling pathway can improve the motor conduction function of the spinal cord, reduce neuronal apoptosis and glial scar formation, and promote axonal growth, so as to promote the recovery of the motor function.

NeuN is a specific protein expressed in the nucleus and cytoplasm of most neurons in the nervous system. It is considered to be a neuron specific marker [33]. This study found that the NeuN protein was less in the model group after spinal cord injury, and the expression of NeuN in the NT-3 and NT-3 + LY364947 groups was significantly higher than that in the model group. After treatment with NT-3 and LY364947, the expression of NeuN protein increased significantly. It is very important to timely and effectively improve the microenvironment of the injured site and induce endogenous neural stem cells to differentiate into neurons after spinal cord injury. NT-3 treatment can reverse the atrophy of most lumbar spinal sensory projection neurons and induce the increase of cell survival in four weeks after spinal cord injury [34]. NT-3 can not only nourish neurons, but also prevent the death of corticospinal neurons induced by axotomy, improve their survival, and induce axon regeneration to restore some of their functions [35]. GFAP is a glial fibrillary acidic protein and a marker of astrocyte activation [36]. The results showed that there was no significant difference in the protein expression of GFAP in each group. NT-3 is a neurotrophic factor required to activate endogenous NSPCs to promote neuronal regeneration and axon remyelination, but this effect is very limited [37]. Most proliferating NSPCs differentiate into astrocytes and fibroblasts to form glial scars [38]. Astrocytes in glial scar are killed or inactivated to weaken their function, which will lead to immune cell infiltration, increased lesion volume, increased neuronal death and deterioration of neural function, suggesting that astrocytes in glial scar play a positive role in the repair of spinal cord injury [39].

After spinal cord injury, TGF-β1 produced and released by astrocytes increases around the injury site, promotes fibroblast proliferation and scar hyperplasia, while the proliferation of reactive astrocyte in the scar forms a physical and chemical barrier to the regeneration of nerve axons and tissue repair, which is not conducive to the treatment of spinal cord injury. Therefore, NT-3 can promote NSPCs to differentiate into neuron-like cells, while LY364947 can inhibit the process of astrocyte promoting scar hyperplasia, but the specific molecular mechanism has not been studied in depth.

After spinal cord injury, some substances that inhibit axon regeneration and cause neuronal cell death will be produced around the injured site. The upregulation of axonal regeneration inhibitory molecules was observed in the injury of central nervous system, such as Col IV, LN, CSPG, tenascin-C, Sema 3A, ephrin-B2 and EphB2 [40]. Compared with the model group, the expression of Col IV, LN, CSPG, tenascin-C, Sema 3A, EphB2 and smad2/3 proteins in the NT-3 and NT-3 + LY364947 groups decreased significantly, while these proteins in the NT-3 + TGF-β1 groups increased significantly compared with that of the NT-3 group. These suggested that the axon inhibitor molecules in tissues can be induced by TGF-β1. When TGF-β1 was antagonized, the expression of these macromolecules decreased significantly. At the same time, it was found that it significantly promoted the extension of neuronal synapses to astrocytes and inhibited the proliferation of fibroblasts [41, 42]. The study showed that the extension of neuronal axons in glial scar could be promoted by regulating TGF-β1.

In subsequent studies, we will further investigate the idea on NT-3 combined with TGF-β signaling pathway activates endogenous NSPCs to regulate their differentiation and promote the repair of spinal cord injury, and use neurosphere assay to determine the differentiation of indicator cells, and Ki-67 immunofluorescence to detect neural cell proliferation. Besides, the myelin g-ratio and apoptotic caspase activation (determined by western blotting or immunofluorescence) may also be explored.

In conclusion, NT-3 combined with TGF-β signaling pathway promotes astrocyte differentiation, reduces axon regeneration inhibitory molecules, reduces apoptosis and glial scar formation, promotes axon regeneration, and improves the therapeutic effect of spinal cord injury repair.

## Data Availability

The datasets used and analyzed during the current study are available from the corresponding author on reasonable request.
